# Deaf people’s experience of the digital transformation of UK healthcare services: a qualitative study using semi-structured interviews

**DOI:** 10.1186/s12913-026-14371-y

**Published:** 2026-04-16

**Authors:** Emmanuel Chan, Sarah Wigham, Amy O’Donnell

**Affiliations:** 1 Mental Health and Deafness Services, Walkergate Park Hospital, Cumbria, Northumberland, Tyne and Wear NHS Foundation Trust, Newcastle upon Tyne, Tyne and Wear NE6 4QD UK; 2 Population Health Sciences Institute, Newcastle University, Newcastle upon Tyne, NE2 4AX UK

**Keywords:** Digital, Mental health, Deaf/deaf, Exclusion, Qualitative

## Abstract

**Background:**

Healthcare services are being transformed with the rapid adoption of digital technology, particularly since the COVID-19 pandemic. However, concerns have been expressed around digital exclusion of potentially vulnerable groups such as those experiencing mental health conditions and/or sensory impairment. This study sought to explore deaf people’s experiences of the digital transformation of UK healthcare services and identify opportunities for future service improvement.

**Method:**

A qualitative study using a social constructionist approach was conducted with data collected using semi-structured interviews. Deaf participants were recruited using purposeful sampling and drawing on existing professional and community networks. Recruitment information was available in British Sign Language (BSL) and plain English formats. Interviews were video recorded, then data transcribed from sign/spoken language to text, with non-verbal and contextual data from signing and social interactions also captured using notations. Video and textual data were analysed in parallel using reflexive thematic analysis informed by the social model of disability and a published framework for evaluating the success of health technologies. Overarching themes across participants’ accounts were identified.

**Results:**

Eleven deaf people participated in the interviews. Ten participants were BSL users; one participant was non-signing and communicated in speech during the interview. Four themes were identified: (1) lack of consideration for deaf individuals’ communication preferences in digital healthcare; (2) social connections facilitate meaningful digital health inclusion; (3) problems with digital systems - flexibility, interoperability and psychological safety; and (4) models of care should optimise deaf people’s independence and accessibility. A study limitation is that data collection and analysis were completed by one researcher.

**Conclusions:**

The findings suggest digital health technology can risk exacerbating health inequalities faced by deaf people. Opportunities for early engagement and involvement of deaf people in designing developing, implementing and evaluating digital health technologies is crucial to ensuring acceptability and services tailored to the cultural, communication, language and literacy needs of deaf communities.

**Supplementary Information:**

The online version contains supplementary material available at 10.1186/s12913-026-14371-y.

## Note on terminology

This article uses the term ‘deaf’ and this is intended to be inclusive to represent the heterogeneity, diversity and intersectional identities of deaf people and communities including people with hearing loss; this terminology/definition is intended to encompass conceptualisations of D/deafness as a socially constructed disability, a cultural identity and as a physical impairment [1–4]. We use the term deaf people rather than person-first terminology to align with more common UK practices [5].

## Introduction

Recent years have seen a rapid growth in the use of digital technology to support healthcare delivery in the United Kingdom (UK) and internationally, particularly since the COVID-19 pandemic [6]. Digital health technologies include smartphone apps, software, online tools for diagnosis, treatment, and health prevention and programmes for analysing healthcare data and improving system efficiencies [7]. Potential benefits of the adoption of health technologies include increased access to personalised, evidence-based care, that is scalable and relatively low-cost [8–10]. In the UK, policymakers have highlighted the potential for digital technology to improve delivery of physical and mental healthcare services through the digital transformation programme [11]. Example initiatives include online self-referral to National Health Service (NHS) talking therapies for anxiety and depression [12] and access to non-urgent medical help via the NHS111 helpline [13].

Despite the potential benefits, concerns have been raised around the impact of the digital transformation of healthcare on health inequalities. Evidence suggests that marginalised groups are at particular risk of facing digital exclusion, such as people experiencing mental ill-health, older adults, second-language English speakers, people with physical disabilities, those experiencing socio-economic deprivation, and those with communication and sensory impairments [14–16]. International research investigating the implementation of digital health technologies among deaf people has found mixed impacts from the perspectives of services users and health services providers. Deaf people reported barriers around usability and convenience of tele-health in the United States [17] and other research has highlighted a knowledge gap regarding the effectiveness of telemedicine for deaf people compared with face-to-face interventions [18]. Evidence suggests digital health technology (e.g. video-conferencing) for staff in specialist deaf mental health services can save time, but highlights obstacles to uptake including limited access to equipment, technical problems and lack of confidence [19, 20].

Other research has found digital health technologies including video relay interpretation to be helpful in overcoming barriers to healthcare access among deaf people [21], and has demonstrated the validity of remote administration of health assessments for deaf people [22]. The launch of the UK BSL999 service in 2022 was received positively by deaf BSL users, with 17,000 BSL calls made in the first 12 months alone [23]. However, questions remain regarding inclusiveness given deaf service users only have direct contact with operators or interpreters but not advisors or clinicians when the call is first made. Further the authors are not aware of any studies of the experiences of UK emergency 999 service users using text-based relay.

Deaf people can experience barriers in accessing primary care through lack of reasonable adjustments e.g. access to a BSL interpreter via video-link [24]. Other research has affirmed this showing that being deaf was associated with less likelihood of accessing primary care or dental appointments if an interpreter was required [25]. In the USA barriers to deaf people accessing digital health technologies included a lack of access to specialist health care interpreters [26]. These experiences may adversely impact the confidence of deaf people in healthcare services, and/or cause feelings of disempowerment and negative emotions [27].

While digital exclusion can relate to availability of technology and equipment, other barriers relate to awareness and training. A study exploring deaf cultural competence among NHS audiology clinic staff, highlighted gaps in staff understanding and expertise regarding BSL users while positive practices included availability of interpreters and staff training [28]. Other contributors to digital exclusion can include lack of personalisation. Research in the USA identified challenges to using digital health technology (mental health apps) for deaf people including lack of personalisation for different language and linguistic needs, a lack of accessible resources, miscommunication and stigma; facilitators included community involvement in app development and opportunities to build relationships with health providers [29]. Another USA study found deaf people who used social media platforms to engage with health information were more likely to communicate electronically with healthcare providers [30].

There are limitations in previous research on deaf people’s experience of digital health technology including a lack of involvement of deaf people in clinical trials, utilizing medical model approaches, inaccessible research methods and lack of meaningful involvement of deaf communities [31, 32]. This study, exploring deaf people’s experiences of the digital transformation of healthcare services in the UK, addressed some of these research gaps by: involving a deaf experts by experience group; being informed by the social model of disability; and focussing on the inclusiveness of digital transformation since the pandemic and best practices for future digital health innovations [33].

## Methodology

We conducted a qualitative study with data collected during semi-structured interviews. A social constructionist epistemological approach [34] informed the methodology and interpretation of data generated from social interactions of deaf participants, their language, lived experience and shared realities to construct a narrative of deaf people’s experience.

The study is reported in accordance with standards for qualitative research (consolidated criteria for reporting qualitative research: COREQ) [35].

### Community and stakeholder involvement and accessibility

Members of UK regional deaf wellbeing networks were invited to join a public advisory Deaf Experts by Experience Group (DEEG) covering the North East UK and North Cumbria. The DEEG comprised 25 active advisors who were deaf or with hearing loss and their supporters, and was aligned with deaf community user involvement best practices [36]. The DEEG was supported by the regional National Institute for Health and Care (NIHR) Applied Research Collaboration (ARC) public involvement and community engagement (PICE) group. The DEEG members were invited to join the group by the researcher EC during monthly online deaf wellbeing network meetings. The DEEG met quarterly throughout the study and contributed to study design, participant materials, recruitment, and sense checking during data analysis to enhance trustworthiness [36, 37]. Meetings were co-led by the first author (EC) and a deaf member of the DEEG, with support from the PICE group manager. DEEG members were reimbursed for their time and travel.

The terms of reference for the DEEG are shown in online supplementary materials (OS1). A second paper will describe development of the DEEG group in more detail.

Professionals from regional deaf healthcare, and community services were consulted regarding the interview process and accessibility.

### Recruitment and data collection

Data collection was completed by one researcher (EC). Purposeful opportunistic sampling [38] was used to recruit deaf adults via regional deaf community networks and social media. Accessible participant information and consent documents including in video format were presented to participants in sign language.

Prior to the interview all participants provided video-recorded informed consent during in-person interviews or on MS Teams.

The interview was presented to participants in sign language or verbally and the topic schedule, demographics form, and interview protocol are shown in OS2-4.

Participants were asked their preferences for a direct interview or with communication support and preferred language (e.g. signing, verbal, or other additional communication requirements); none requested an interpreter, the majority preferred signing with one deaf non-signer participant opting for speech with visual cues. All participants were interviewed directly by EC one-to-one (*n* = 7) or in dyads (*n* = 4), where interview questions were presented to two deaf people simultaneously allowing interviewee discussion and observations of their interactions (by EC) [39].

Participants were offered a pre-interview opportunity to ask questions, test the online platform and online or face-to-face interviews at home or a community venue. Three participants preferred online and were interviewed individually via Microsoft Teams. The other interviewees including the dyads were interviewed in person. Interviews were video recorded, anonymised and transcribed into written text via repeated viewing by EC.

Bi-directional interaction with interviewees and the interviewer asking questions for elaboration and examples to check meaning and interpretation, helped optimise interview dialogue, transcription accuracy and trustworthiness [40].

Interview participants were renumerated and offered a follow-up wellbeing interview; while no interviewees requested follow-up, six continued DEEG participation.

### Data processing and analysis

#### Reflexive analysis

Reflexive thematic analysis was used [41–44], as in previous research analysing data collected from research participants using sign language and video [45–47]. Reflexive thematic analysis provided a means of iteratively exploring participants’ experiences with digital health technology while acknowledging the researcher’s viewpoint as active and integral [43, 44].

#### Transcription and reflexivity

Researchers with deaf communities have proposed a ‘paradigm shift’ in deaf qualitative research with analysis applied directly to signing video data during the process of transcription [32]. This method can help reflexivity with researchers considering whose interpretations are reflected in analysis: the participants, the researcher, or a combination of both [32].

There are epistemological challenges to consider in transcribing data from one language into another including that BSL is a visual language, does not have a written form and is not the transcribers first language [48, 49]. The decision to transcribe from BSL into written format was pragmatic, influenced by plans to disseminate the research findings to clinicians, service providers and developers who may be able to instigate change to health services but may not be familiar with BSL. Transcription and analysis were completed by EC with semantics and prosody of the social dialogue studied to explore stories shared by participants.

#### Coding and theme development

Data analysis, was completed by hand and iterative, with written transcripts revised and edited alongside viewing the videos, using the framework method to organise video data, alongside reflections and annotations in the transcripts [50, 51]. An initial framework with codes and themes was generated by the researcher (EC), for the purpose of organising data then shared with members of the DEEG group for sense-checking and subsequently discussed and developed with the wider research team over several meetings. Revisions were made to the framework, codes and themes based on feedback from the DEEG and the research team. The process of data coding and revising and developing themes was iterative. Data were reviewed repeatedly to capture the subtlety of participants’ responses. This involved examining and coding non-verbal cues and non-manual features for example pauses, intonation, hand shapes, body movements, postures and facial expressions (e.g. raised or dropped eyebrows) [52]. Themes were created from collating similar codes.

#### Theoretical frameworks

Data analysis was informed by a framework relevant to predicting and evaluating the success of digital health technology (non-adoption, abandonment, scale-up, spread and sustainability: NASSS) [53, 54]. Analysis was also informed by the social model of disability highlighting how social structures can exacerbate or mitigate health inequalities [55] with interpretation of participants’ views guided by perspectives on barriers and exclusions [33]. These approaches helped explore barriers and facilitators to acceptance/non-acceptance of digital health technologies and wider societal factors [33, 54].

Further detail on the methods is shown in OS5.

### Positionality and reflexivity

EC is a hearing mental health professional with advanced level signing and communication competencies who has worked closely with deaf communities for over thirty years, which has facilitated ongoing partnerships, and established trust and familiarity [36, 49]. EC is a member of a minority ethnic group and experienced in transcultural mental health issues related to culture, identity, language and literacy and the impacts of exclusion and discrimination.

It is important for hearing researchers working with deaf people to contextualize and reflect on their position within socio-political structures [56]. EC reflexively considered his social, professional and cultural positions during data collection and analysis and used self-inquiry to reflect on his influence on research processes and to avoid assumptions. EC was supported by lived experience experts (the DEEG), health practitioners and academics with expertise in qualitative health services research and this facilitated accessing diverse perspectives strengthening trustworthiness of the findings [37].

The study was approved by Newcastle University Faculty of Medicine Ethics Committee (NCL reference 27383/2022).

## Results

Eleven deaf people participated in the study, an appropriate number for reaching saturation in qualitative research [57]; all were over 45 years of age (Table 1). Ten participants were culturally deaf and fluent signers including one participant, a recent migrant, who had only used BSL since arriving in the UK two years ago; one participant was non-signing and communicated in speech during the interview. All participants were born with hearing loss and identified as having disabilities; seven identified with sensory impairment (i.e. deafness); one self-reported deafness and mental ill health; two both deafness and long-term health conditions; and one participant self-reported deafness and physical disability. Most participants had social contact with other members of deaf or hearing loss communities and received formal or informal support from them. Interviewees experience of using technology is shown in Table 1. The interviews lasted 90 min on average.

**Table 1:** Interview participants characteristics

Participant	Description
S1	White male. Age 70–80 years. Married. Born deaf and signer. Active in deaf communities. Retired. Experiencing long-term health and mobility problems. Used technology.
S2	White female. Age 70–80 years. Married. Born deaf and signer. Active in deaf communities. Retired. Deaf sibling. Used technology more during COVID.
S3	White female. Age 40–50 years. Single and living alone. Born hearing. Prelingual deaf/signer. Active in deaf communities. Unemployed. Experiencing long-term health problems. Used technology.
S4	White male. Age 60–70 years. Married. Born deaf and signer. Active in deaf communities. Retired. Experiencing long-term health and mobility problems. Relied on family for technology.
S5	White male. Age 60–70 years. Married. Born deaf and signer. Active in deaf communities. Retired. Used technology.
S6	White female. Age 60–70 years. Married. Born deaf and signer. Active in deaf communities. Retired. Not keen on technology.
S7	White female. Age 40–50 years. Partnered. Born deaf and signer. Not active in deaf communities. Employed. Experiencing long-term health problems. Used technology but not keen.
S8	White (other) male. Age 30–40 years. Single living with family. Born deaf abroad and multilingual signer. Active in deaf communities. Employed. Used technology regularly and interested to share with peers.
S9	White male. Age 30–40 years. Single and lived alone. Born deaf and signer. Active in deaf communities. Unemployed. Experienced long term health problems. Used technology regularly and interested to share with peers.
S10	White female. Age 60–70 years. Single. Born deaf and non-signer. A bit active in deaf communities. Retired. Experiencing long-term health conditions. Interested in technology but not confident.
S11	White female. Aged 40–50 years. Single. Born deaf and signer. Active in deaf communities. Unemployed. Experiencing multiple long-term health conditions. Interested in technology but not confident.

### Key themes

The initial framework matrix with codes linked to each theme are shown in supplementary materials (OS6); this framework which developed starting at the individual level and advanced to the organisational macro level was revised iteratively during analysis leading to the development of four final overarching themes. These are described in the text below and include: Theme 1: Lack of consideration for deaf individuals’ communication preferences in digital healthcare; Theme 2: Social connections facilitate meaningful digital health inclusion; Theme 3: Problems with digital systems flexibility, interoperability and psychological safety; Theme 4: Models of care should optimise deaf people’s independence and accessibility.

## Theme 1: Lack of consideration for deaf individuals’ communication preferences in digital healthcare

Some participants described experiencing various challenges growing up, often as the only deaf person in their family, and all reported having accessed special education for deaf people. Most recalled being moved from mainstream to special schools for deaf people or for people with additional educational needs, and frequently experienced bullying alongside separation anxiety from their parents and siblings. Despite attending specialist schools, many participants reported that education provision was not often tailored to their communication and education needs rather focussed on listening and speaking (oralism). This oral English-based teaching environment and early disruptive education experiences appeared to be perceived as having adversely affected some participants’ general literacy and capacity to acquire new knowledge and skills, including potentially the use of digital health technology:

*I missed out a lot at school or at work. People laughed at me because I was not able to pick up what they said…how I was treated at school has been affecting me now. I have no confidence. When I saw doctor*,* I just said yeah*,* yeah*,* yeah (sighed and head shaking). (S10)*

Some participants’ felt that adverse educational experiences were replicated later in life during interactions and engagement with digital health technology and service providers. For example, although it was clearly important to most participants that healthcare providers tailor communication to meet individual needs and preferences (most commonly, signing, using visual learning and cues) this was rarely available in digital health technology spaces.

Access to an interpreter was viewed by some participants as essential in appointments with healthcare professionals, however, only a few reported being offered online interpreting or VRS (video relay services). Even when interpreters were available, some participants described additional challenges including professionals being unfamiliar with wider communication tactics (e.g. ‘*looking at me’*, ‘*setting the scene’*, or ‘*recap and summarise’)* and regional differences in sign vocabulary:

*At times I was struggling with the interpreters’ signing as they might come from other parts of UK and used other regional signing vocabularies. I got confused a few times with the variations in numbers*,* days of the week and some specific signs. It was quite funny. I had to check with the interpreters a few times for clarification (S7)*

Some participants perceived a need for personalised training for deaf people tailored to individual learning styles and language and delivered by familiar/trusted professionals to enable meaningful participation in digital health technology:

*Better to have a course especially for older people. We can learn a little every week - not too much*,* just a small dose’ (nodding and smiling) (S6)*

Some participants mentioned finding BSL videos unhelpful including describing signing as too fast and closed captioning language too complicated. In contrast some participants were impressed by improved access to emergency services through digital health technology having installed apps or devices at home to access services such as fire, ambulance and police and reported positive experiences in contact with them.

## Theme 2: Social connections facilitate meaningful digital health inclusion

In the pandemic, deaf people’s access to peer support significantly changed when in-person centres and clubs were closed during lockdown restrictions. Some participants with multiple long-term health conditions recalled being classed as vulnerable, and were told to stay home and shield. Some recalled attempting to maintain contact with friends and families by forming ‘bubbles’ in accordance with official social-distancing guidance. Others described using online video platforms such as Facetime, WhatsApp, and Zoom to maintain social contact. Some participants believed deaf communities became more dispersed post-pandemic, with several deaf clubs remaining closed or merging with wider community hubs.

Some individuals continued to use online platforms to maintain social connections, but did not appear to find these as fulfilling as face-to-face meetings; dissatisfaction with poor connectivity and limited device battery life was reported:

*We are aware of online meeting with other deaf people during Covid-19. I preferred to see other deaf face to face. We go one deaf club every Thursday if they can for a change of scene from home… the open space is important to us. (S2)*

Some participants also highlighted the manners and etiquette of online meetings as inconsistent with deaf culture: signing in-person is three dimensional, and watching signers online reduces the dimension of depth. Others commented that behaviours such as interrupting another deaf person, pointing to someone and prompt questioning could be difficult.

Others noticed that while more deaf peers were using online social platforms *‘having a good natter in the same room with their friends’* would still be better as sign language is a visual spatial language, therefore meeting online might not be as good.

Most participants expressed a desire to exercise control and choice about digital health technology, but often felt ill-equipped to do so, instead relying on families and friends for assistance.

*I usually ask my sister for downloading the app. I was not sure about my log in or password. My nephew does most for me and help me to log in. I have got them written down on my little book. I don’t like dealing with my password or going to the ATM [bank automated teller machine] hole in the wall. My nephew set up my broadband and a few more things on my TV’ (relaxing shoulders and phew lips as signs of relief) (S10)*

Several participants with multiple long-term health conditions highlighted that without peer support to help meaningfully engage with care providers, digital health technology could be challenging. Families, friends, and the deaf centres were very important to providing support to make hospital calls, ask for interpreters, or to set up remote consultations:

*I have heard of [deaf-led charity] but am not sure about their services such as the BSL health information library or how to navigate. I try to learn on web*,* look for information and it is not always easy being on my own as some English words are hard for me. (S11)*

In contrast some with support to use digital health technology from hearing family members or friends described challenges in having to share personal information and communicating needs, partly due to a fear of being seen to lose control or show weakness, and some expressed deference to the views/opinions of hearing carers: ‘*hearing people know more and know best’*.

### Theme 3: Problems with digital systems flexibility, interoperability and psychological safety

Some participants described not having the skills or not feeling safe with digital health technology, also that some healthcare services do not always have positive attitudes towards deaf people, and/or lacked knowledge and competency in digital health technology impacting their willingness to use it. Some participants described being told about digital health technology service changes without opportunity to express their view, and that services and information were not always accessible. In contrast participants who were more digitally curious and confident had asked about digital health technology such as video relay interpreting, remote consultation, and access to their healthcare records and some were pleased with the responsiveness and flexibility of providers. Others described disappointment when told that digital health technology e.g. video relay interpreting or remote consultation could not be provided. Others conveyed frustration that interpretation services did not meet quality requirements for accessibility:

*I visit hospitals regularly for my health problems. I only find out who the interpreter is when I turn up my appointment. I am not happy about this (eyes open wider*,* eyebrows raised). The service should respect my preference and inform me in advance the details of my communication support. (head nodding a few times) (S11)*

Most participants reported feeling that their health needs were not met during the COVID-19 pandemic, in part due to rapid moves to telephone-based care provision. Participants reported relying on hearing family or friends to contact healthcare services to inform them that telephone appointments would not work because they are deaf. Some interviewees described this as frustrating, as it suggested a lack of attention to information previously provided for electronic medical records regarding their deafness, communication and information requirements. Some questioned why their accessibility needs were not replicated between health record systems so that they did not have to repeat communication preferences:

‘*No reply’ messages from doctor or hospital…. I don’t always understand and cannot reply to tell them that the messages are not making sense. Or allow me to confirm or cancel the appointment or ask if an interpreter is booked. (S6)*

Some shared frustrating experiences of healthcare transitions e.g. between hospital and primary care and a lack of communication between services about aftercare and indicated that digital health technology might not improve this. One participant recalled being given a paper before leaving hospital but not knowing if it was a discharge letter or follow-up arrangements:

*A nurse should visit me to check my foot operation and change my dressing…nobody came. I decided to go back to the hospital ward and sort it out myself. It is not acceptable. (S5)*

Some participants reported that available digital health technology was often not user-friendly. Some participants were familiar with the NHS app and knew it could be used to book GP appointments or order repeat prescriptions or vaccines. However, some participants who had used the NHS app, reported it taking too long and involved too many steps to carry out relatively simple requests, such as communicating with their GP. It was perceived to be easier to turn up at the GP practice in person. Further, whilst most participants were aware of mental health listening or round the clock emotional text messaging platforms such as ‘Text Shout’, they were disappointed by long delays in receiving a reply:

*I used NHS app and answered the questions*,* but I had to wait for an hour and did not get any reply. I really wanted to have some medications as quick as possible. I tried messaging and using the app*,* I had to wait. It was frustrating and making me feel lost. I had to ask my sister to ring the doctor*,* and she got through quickly and got me what I wanted. It was so different. (S8)*

While all participants had access to the internet some reported concerns with the age and specifications of their personal devices, and that they tended to choose devices and internet providers based on costs and connectivity. Some were aware of special lower cost packages for people on welfare and benefits, but these often failed to meet their requirements, e.g. available devices and networks were not reliable and fast enough for videocalls. All participants reported feeling dissatisfied and demotivated when the connection was interrupted or disconnected and felt more reassured by in-person meetings as a result:

*I am struggling to connect my equipment with the Wi-Fi. I tried messaging them via WhatsApp to set up but found it not easy. Cost is a factor in my decision for broadband package. I have to be careful with my spending. I signed a contract with a provider for 18 months…feeling stuck. (Looked dissatisfied) (S11)*

Most participants expressed feelings of confusion and disorientation in the digital health technology world. Many reported downloading several apps onto their phone during the pandemic either advised by their doctors or nurses or family members but did not feel confident to use them ‘*they are just there on my phone*’ Various digital health technology concepts and terms were discussed during the interviews (e.g. *patient portal*,* remote access*,* video relay*,* videocall*,* biometric login)*, however many participants tended to reply that they had ‘no ideas’ to most of them. Cybercrime was perceived to have undermined many participants’ sense of safety around using digital health technology. Most participants reported experiencing scams and suspicious emails and messages. Some had made mistakes, suffered financial losses, and felt uncertain and anxious about receiving messages or emails and often just deleted them including genuine ones from healthcare providers. Most struggled with software updates and installation especially those with older devices.

### Theme 4: Models of care should optimise deaf people’s independence and accessibility

Participants highlighted that deaf people have been exposed to technological equipment and devices since they were born. For example, hearing aids and cochlear implants, radio aids at school, minicom/textphone, flashing lights or pagers (linked to the doorbell, telephone, smoke detectors and baby alarms), blue tooth technology between portable loops, phone and television and care-call pendants. All participants agreed that text messaging and app-based communication via their phone were the top two applications they used in day-to-day life. iPad or hand-held devices had become more popular during the pandemic and were viewed as good for videocalls with families and friends via WhatsApp and Facebook. All participants reported having installed a variety of apps on their smartphone for functions ranging from instant messaging, health and wellbeing, access to urgent and emergency care, communication and video/text relay, banking, plus for transport, shopping and directions.

Participants described how the offer of digital health technology with reasonable adjustments introduced and explained in a positive way by confident staff was helpful:

*My interpreter did not turn up. The reception staff apologised and suggested video interpreting and asked if I wanted to have a go. We never had the support before and said fine. We were accompanied to a room with a big TV. Shortly after*,* someone popped up on the TV – a BSL interpreter. The screen was big and the image was clear. We were impressed. (smiling and praising look) (S1)*

Some participants reported becoming familiar with the NHS119 vaccination booking app during the pandemic and the direct link to video relay was praised for respecting communication and language needs. Others used the NHS111 phone number but appeared to find the experience more haphazard as their needs were not met in a timely manner.

Access to emergency care was also highlighted with some participants reporting being familiar with Relay UK (or previously the NGT (next generation text) relay service) and using the BSL999 number when it was launched during the pandemic. Most reported positive experiences of these and feeling able to access services round the clock in emergencies. Older participants reiterated the benefits during the pandemic when they had to call for help due to Covid-19 infections and falls, had prompt responses, and felt interactions with call-handlers provided them with reassurance and comfort. One participant was introduced to remote patient monitoring from home during the pandemic for heart rate, blood pressure and oxygen saturation and elaborated on differences in her experiences of services from her GP and ambulance service:

*When I caught covid*,* the surgery sent me some medication and a device to check my blood. I did not know how to use it until a lot later. It was an oximeter (got its name much later). The surgery said they would contact me to explain but they never did. I called NHS111 and the ambulance people came quickly. It was alright as they explained to me how to use them patiently. The face-to-face contact and their patience meant a lot to me. (S10)*

For some participants benefits of digital health technology included the potential to promote and facilitate independence and safety. These participants reported using web-based information and online groups set up by and for deaf people and found them accessible and helpful in understanding their health issues. They were impressed by the improved access to the emergency services. Most had apps installed or devices at home to access services such as fire, ambulance and police and reported positive experiences when in contact with them during the pandemic. Some participants preferred using the Relay UK services while others had familiarised themselves with the BSL999 app.

Other participants, often younger and male, were also positive about using digital health technology and conveyed a sense of validation from having the opportunity to manage their own care. These individuals appeared to embrace available digital health technologies such as the NHS and BSL Health Access apps which allowed them to book appointments, order repeat prescriptions, and use video relay services to access their GPs and other NHS services. For example:

*I’ve got smartphone*,* laptop and iPad and all linked to my internet. I use NHS app to order my prescription and check text results. It is very good. I think my internet skills and knowledge was 8 out of 10. (face of pride) (S9)*

This contrasted to the views of other participants who felt they lacked skills and had safety fears e.g. of scams and described feeling overwhelmed by for example the number of apps available which could be anxiety provoking.

## Discussion

### Key findings

This study explored deaf people’s experiences of the digital transformation of health and care services in the UK. It aimed to understand the extent to which digital transformation has been inclusive and to identify best practices in delivering future digital health technology innovations for deaf people. Similar to previous research [19, 20] a key study finding is that most participants commonly experienced challenges with using or accessing digital health technologies in healthcare settings. For example, due to complicated closed captioning, difficulties watching signers on screens as compared to in-person, or unavailability of video relay interpreting.

While some participants viewed digital health technology as occasionally helpful it did not always facilitate independence or feelings of empowerment. Participants in previous research have described limitations with interpreter support, highlighting that training is required for remote interpreters in healthcare e.g. on medical terminology [26, 58]. Participants in the current study similarly reported that reasonable adjustments were not always available, limiting meaningful and/or effective engagement with digital health technology. This is concerning given higher rates of mental and physical health problems experienced by deaf people and barriers to accessing care [59]. Previous research has identified that although anxiety and depression are higher among deaf people than the general population, few access NHS Talking Therapies [60].

We found that reasonable adjustments, accessible communication, and information requirements were not always transferred between different health services record systems, causing frustration for service users. This could be due to fragmented electronic patient records systems, restrictive information sharing between providers [61–63] and/or practitioners having limited understanding of how to provide reasonable adjustments [64]. Limited understanding of reasonable adjustments has been highlighted among practitioners working with other groups facing healthcare access inequalities including autistic people and people with a learning disability [65–67].

Existing literature shows that deaf people face inequality in accessing healthcare services [59, 68, 69]. Likewise, participants in this study highlighted widening health services inequalities for deaf people post-pandemic due to reduced community support (e.g. deaf centre closures) and reduced local authority funding, and indicated that digital health technology challenges could compound these inequalities. However while most participants highlighted problems with digital health technology, a few participants found some technologies were relatively low cost [8–10] and were able to add for example health apps onto their phones with relative ease.

### Barriers to adoption of digital health technologies

Our study found a key challenge to adoption of digital health technologies by deaf people was needing to rely on family or friends to support access, potentially compromising independence and privacy. Concerns with cybersecurity further heightened feelings of psychological vulnerability and risk and compromised willingness to adopt digital health technologies.

Participants implied experiencing discrimination through health services inequality, was traumatic and such negative experiences could significantly adversely impact future acceptance of digital health technology among deaf individuals and communities. Many participants described emotions including anger, frustration, loss and confusion at some of their experiences with digital health technology. Research shows increased risk of language deprivation, and unique early traumas among deaf people compared to hearing populations [70–73]. Participants in this study suggested that feelings of exclusion through early adverse childhood and communication experiences were intensified when digital health technologies were imposed or inaccessible. Some participants reported that health care staff had secured their trust and helped sustain their adoption of digital health technologies and the findings highlight that implementation of digital health technology with deaf communities should be compassion and safety informed [74–76].

Problems with personalisation, user-friendliness, accessibility, the quality of pixelated effects and live transcriptions were frequent examples reported by participants of barriers to adoption and factors in abandonment of digital health technologies. Similar to previous research highlighting limited involvement of deaf people in research [31, 32] participants raised questions about whether digital health technologies were sufficiently tested, reviewed, and valued by deaf users prior to implementation.

### Facilitators to adoption of digital technologies

Participants were positive about the NHS119 and BSL999 apps with previous research reporting high numbers of callers to the BSL999 service [23]. Participants were curious about digital health technology when offered support to prepare for implementation, and where healthcare workforces and organisations were adequately equipped to accommodate personalised adjustments. Examples of adjustments described as helpful for alleviating disadvantage and uncertainty included co-created resources, individualised training (for deaf people and service providers), and accessible health promotion materials. The findings suggest training and support for deaf people to use digital health technologies and training and deaf awareness for staff should be sustainable (not one-off) and co-created, with involvement of deaf lived experience experts.

Previous research highlights co-designing/co-developing [4, 77, 78] digital health technology as essential to successful digital transformation, enabling users’ empowerment, and ensuring appropriateness and acceptability of technologies. The study findings indicate the importance of early engagement with deaf communities as co-creators so that barriers can be mitigated, managed and addressed. Digital health technology benefits may be discounted by deaf service users unless user feedback is incorporated, personal contexts such as culture and identities are considered sensitively, and societal preconceptions of deaf communities challenged [4, 32, 77, 79].

### Study strengths

Deaf communities are often excluded from meaningful engagement in research [31] and researchers may lack deaf awareness and a study strength is focussing on the experiences of deaf people, and how their unique individual identities interact with digital health technology. A further strength is involvement of the DEEG group throughout the study; this was informed by previous research on user involvement e.g. that the researcher be familiar with deaf communities and BSL and the importance of ongoing partnerships [36].

### Limitations

It cannot be assumed that the views expressed here would be the same had another or more extensive cohort been interviewed; however a small sample is not unusual for qualitative research [57]. All participants were white, all resided in one specific UK region, and most were over fifty years of age with additional health and social challenges. Older adults have been identified as particularly at risk for digital exclusion [80]. Some older participants we interviewed felt ambivalent or reluctant to use digital health technology after experiencing cybercrime or scams, and digital literacy limitations may also have been a barrier in the rapid pace of digitalisation. A younger cohort may have expressed different views as previous research found younger deaf people who use social media more likely to communicate with health providers electronically [30]. The views of participants may therefore not be representative of deaf people with other demographic characteristics, including other ethnic and cultural groups, and this is an important area for future research.

One researcher (EC) completed data collection, transcription and analysis, albeit with input from the DEEG and academic co-authors and this potentially introduced bias. Independent transcribers and interviewers with lived experience could have improved impartiality; however, a single researcher facilitated deeper engagement with participant data. A further limitation is the absence of a deaf researcher in the team, and while some parenthetical notations were included with quotes to reflect the richness of visual information conveyed by participants a formal notation system was not used.

### Policy and practice implications

While some participants showed interest in using digital health technology, there was a sense of injustice that developments remained focussed on hearing people. The findings highlight that digital health technology innovation must be informed by the experiences and preferences of deaf people, and the importance of early communication and support for deaf service users when implementing new digital health technologies. It is important to consider the complex healthcare service contexts and policies influencing acceptance of digital health technology, and the insights learned from situations where digital health technologies were abandoned or dismissed can inform organisations completing digital maturity assessments [53, 81]. Identifying gaps in satisfying digital health technology service accessibility requirements for deaf people is key to addressing health inequalities faced by this group [81].

The findings highlight the need for digital health technology developments to consider the diversity and heterogeneity of the deaf population and the social, organisational and interoperability barriers they may face. Further the findings highlight the importance of comprehensive workforce preparation through training, digital skills assessment and deaf awareness raising regarding platforms designed to include people who are deaf. For example the acceptability of video-relay to deaf people was shown to be contingent upon availability of interpreters trained in medical terminology [21]. Deaf awareness training and reflection on accessibility practice can facilitate questioning service providers’ assumptions [28, 82]. Training could include deaf-led digital awareness and safety sessions, be co-created training and integrated with under/post graduate healthcare practitioners’ and continuing professional development (CPD) opportunities. This would align with the social model of disability, with changes made at systems and organisational level [33].

Participants highlighted that support should be personalised matching individual language, cultural and communication needs and preferences; continuity of care both of practitioners and interpreters could help ensure engaging with providers who are aware of individual communication methods and requirements and reasonable adjustments.

### Future research

There have been recommendations around system wide changes such as better commissioning arrangements for BSL interpreters and other communication professionals, workforce training, and deaf communities’ engagement [28, 83–87].

Implementation science and realist approaches [88, 89] could be used to help to understand barriers and facilitators to the effectiveness and acceptability of digital health technologies across different organisational systems which were identified as a problem in this study. Research into methods of personalisation of digital health technology is key given the heterogeneity of deaf communities e.g. for the specific support needs of those deaf people who are carers of people with dementia and the challenges faced by them during the pandemic [90]. Some of the unique challenges faced by older people in China during the pandemic highlight the need to consider how such challenges might be mitigated in any future international health crisis including focussed bolstering of emergency communications [91].

Much digital health technology has been developed ‘for’ deaf people by mostly hearing researchers and future research should focus on user-centred design that facilitates deaf people influencing innovations [4, 92]. This could be informed by the characteristics of digital health technologies that have shown good acceptability e.g. BSL999 [23] and those specific features e.g. of mental health apps that are identified by members of deaf communities as facilitators to adoption [29].

Future research could compare concordance/compliance with clinical interventions [93] with adoption/abandonment of digital health technology by deaf people e.g. adverse communication experiences, psychological safety and digitally related trauma. Further exploration is required on accessibility of digital health technologies for other underserved deaf groups e.g. people with a learning disability. Future research should examine the interaction of adoption/non-adoption of digital health technologies among deaf people with implementation of ongoing NHS policies especially the areas covered by the NHS Long Term plan from hospital to community, from treatment to prevention, and from analogue to digital [94]. Finally how digital and health literacies among deaf people will interact with the success of accessible information and reasonable adjustments requirements in the further transformation agendas within health and care services.

A summary of recommendations for future research includes: (1) in-reach to less visible members of deaf communities; (2) evaluation of how deaf people could be invited to and sustainably represented as part of PICE initiatives such as the DEEG; (3) evaluation of the effectiveness of organisations in meeting accessible information and reasonable adjustment requirements; and (4) enhancing systems for personalised care and self-navigation within electronic patient records e.g. digital hospital and communication passports [95].

## Conclusion

Significant organisational and personal barriers to successful engagement with digital health technologies by deaf people have been identified in the study. The study findings suggest the barriers to accessing health services faced by deaf people can be cumulative; some study participants had co-occurring health conditions and people with health conditions and disabilities are more likely to be digitally excluded [96]. The findings highlight how digital health technology in healthcare can risk further marginalising deaf people and how barriers to engagement with digital health technologies can compound/exacerbate pre-existing health inequalities.

These are crucial to address given the increased reliance on digital health technologies of deaf people e.g. video relay compared with the general population. Provision of accessible information, reasonable adjustments and relevant training for deaf people and service providers are important. Involvement of commissioners and policy makers who would have the leverage to instigate macro level changes is crucial as are empowering approaches acknowledging the strengths and resilience of deaf people (e.g. familiarity with limited social contact) and positive deaf cultural identities [5, 97–99]. Early engagement and involvement of people with lived experience throughout the design, development, implementation and evaluation of digital health innovations is key to ensuring new technologies are acceptable, fit for purpose and to facilitate adoption, scaling up, and sustainability of digital health technologies for deaf communities.

## Supplementary Information

Below is the link to the electronic supplementary material.

Supplementary Material 1

## Data Availability

The datasets generated and/or analysed during the current study are not publicly available due to confidentiality but are available from the corresponding author on reasonable request.
