# Supplementary material for: Deaf people’s experience of the digital transformation of UK healthcare services: a qualitative study using semi-structured interviews
Source: BMC Health Serv Res. 2026 Apr 16;26:753. doi: 10.1186/s12913-026-14371-y (PMC13202999; doi:10.1186/s12913-026-14371-y)
Supplement: Supplementary file 1 — Supplementary Material 1 [file 12913_2026_14371_MOESM1_ESM.docx]

**Online Supplementary Materials**

**Online Supplementary Materials 1 (OS1):** Deaf wellbeing network/experts by experience group terms of reference

**TERMS OF REFERENCE (TORS) OF NORTHEAST DEAF EXPERTS BY EXPERIENCE GROUP (DEEG) IN RESEARCH**

**What we do?**

The terms of reference or TORs is to get your support in research.

We call you Deaf Experts by Experience DEE. The Group DEEG in Research is part of the work by the Northeast and North Cumbria NIHR ARC (National Institute for the Health and Care Research Applied Research Collaboration).

**Why you?**

You are Deaf, deaf, hearing impaired or deafblind with lived experience. We use deaf to describe all of you below to keep the names shorter.

Some of you are carers, family members or friends who support deaf people.

You have some experience in using health and care services and technology and their barriers.

You want to improve the services for deaf people and make sure the services are accessible and fair. We call you “Experts by Experience” in research.

You would like to work together with other deaf experts and their supporters.

You represent different people from the deaf communities. We call this “Diversity”.

**What are our aims?**

We want to find out from you

1. how deaf people can feel safe and supported when taking part in research.
2. how deaf people can take part in research comfortably and confidently
3. if deaf people can understand the research information and questions
4. how the data can be analysed and understood
5. how the results can be concluded and shared with deaf communities
6. what should be done to improve the health and care services after the research
7. how do we know if it is successful or not

**Who you are?**

The DEEG group is open to people who are Deaf, deaf, hearing impaired and deafblind. Some of you will be

- Black or Asian or from other races and/or
- Lesbian, Gay, Bisexual, Transgender or non-binary.
- Of different age groups
- Living in rural or city areas
- Married, partnered, divorced or single

All above are invited to be part of the Group so that you are represented. We call this “inclusive”.

**What you do and offer to the Group?**

1. To work in a group and respect each other and their differences
2. To listen to each other and follow the agreed rules and manners of communication within the deaf communities
3. To agree and follow the agreed ground rules and behaviours so that the Group can work together productively with respect for each other.
4. To attend some training and meetings face to face or online
5. To get to know the research topic “Deaf people’s experience in Digital technology”
6. To provide your views and share your lived experience
7. To come up with ideas in research or other topics
8. To consider taking up researcher roles in the future if you want to.

**What you get in return?**

1. You will be offered payment and recognised for your time and traveling
2. You will be provided communication and other support required by you.
3. You can access training and support in working together and research
4. You can attend other training provided by the NIHR ARC
5. You can be supported to become a researcher and apply for funding.

**What are the ground rules for working together?**

1. Remember the fundamentals for successful communication such as clear, correct, complete, concise, and compassionate. They might vary! Everyone accepts individuals’ identities and communication preferences We review and make continuous adjustments depending on circumstances and changes.
2. Follow the communication tactics with people who are deaf or with hearing loss. Respect their preferred communication methods. Everyone’s preference is recognised.
3. Respect each other and their values and beliefs. Everyone’s opinion matters.
4. Maintain confidentiality and privacy. Everyone is responsible.
5. There are no stupid questions. Everyone’s voice is heard.
6. Actions and words and signs should all match. Everyone learns from each other. We are role models to each other and share our responsibility to lead.
7. Make time to prepare before the meeting. Briefing and debriefing is important to everyone (deaf, hearing members and communication professionals) to make the meeting supportive and productive.

**Online Supplementary Materials 2 (OS2)**

**RESEARCH STUDY: Deaf Digital Transformation Experience Study**

**PARTICIPANTS PERSONAL DETAILS FORM (“ABOUT YOU”)**

|  |
| --- |
| 1. What is your gender?   Male Female Prefer not to say |
| 1. Is this the gender you were assigned at birth?   Yes No Prefer not to say |
| 1. What age are you?   18-29 30-44 45-64 over 65 Prefer not to say |
| 1. How would you describe your ethnicity?   White/White British Mixed/Multiple ethnic groups Asian/Asian British  BlackAfrican/Caribbean/Black British Other ethnic groups Prefer not to say |
| 1. Do you consider yourself to have a disability or long-term condition?   Yes No Prefer not to say |
| 1. If yes, how would you describe your disability?   Physical disability Mental health related disability Learning disability  Sensory impairments long-term health condition  Prefer not to say |
| 1. What is your employment?   Employed Unemployed full/part time education  Long-term sick/disabled Looking after family/not working/seeking work  Unpaid voluntary work Retired Prefer not to say |
| 1. What is your preferred communication support?   BSL SSE Lipreading Speech to Text translation Others |
| 1. What is your preferred contact method?   Videocall Text messaging Email Voicecall Post Minicom others |
| 1. What is your postcode? __________________ |

**Online Supplementary Materials 3 (OS3). Deaf Digital Transformation Experience Study**

**Participant Interview Topic Guide**

**Introduction**

- Thank you for attending the interview/group and agreeing to share your views about
  - digital changes in health and care before,
  - during and after the covid19 pandemic as a deaf person.
- Anything you share today will be treated with confidence. Information you share will be kept private and secured and your personal details can only be identified by people working with the project; NOT MEMBERS OF THE DEAF COMMUNITIES.
- Can I check that you have read and understood the participant information sheet and/or the explanation again in BSL. Please let me know if you have any questions for me?
- You might find some signs or words not familiar. We will go through them quickly now and again during the interview
  - Digital
  - Transformation
  - Your feedback
  - Remote access (seeing professionals/getting help) from home or not at hospital or surgery
  - Video consultation
  - Web-based information
  - Apps-based support – BSL999, NHS111, BSL health access/InterpretNow/Signvideo
  - Cybersecurity and personal safety e.g. risk of scam
  - And many more
- I will now go through the consent with you. It is a written document supported by BSL to make sure you understand and agree with the interview today.
- You have a right to answer or not to answer any questions. You can also decide if you want to stop the interview or take a break.
- I thank you for your support for this study. I fully respect your answers as they are your views. There are no right or wrong replies.
- Are you ok for me to turn the video camera on and start the interview?

**Background**

First, we would like to find out a little about you and your situation.

1. Please could tell me a bit about yourself and your contact with other D/deaf people? (probe: background of deafness, preferred communication and support, social/familial support e.g. other D/deaf family members and access to D/deaf groups, employment)

As you know the aim of the study is to understand Deaf people’s experience of digital changes in services and care in NHS and if you have useful or not useful; and/or improved your health and wellbeing.

It would be helpful to understand a bit about the background of your health and your contact with health and care services. We will talk more about your experience of digital technology and information later.

1. Please could you tell me about your health and your experience of using health and care services as a Deaf person? (probe: physical and mental health conditions, access to interpreting and translation support, how they affect your life and living?)

**Accessing digital health and support**

We are interested in Deaf people and their use of technologies and information such as video-consultation, websites and self-help apps

to access health and care services;

before, during, and after the main covid19 pandemic between 2020 and 2022.

Understanding of the Digital Infrastructure and connectivity.

When talking about infrastructure and connectivity, we would like you to think about your experience with

- digital equipment, devices, and
- connection to the internet at home or elsewhere e.g. public library, Deaf centres and
- how easy they are to get to and be connected. (Accessibility)

1. Could you tell me about:
   1. what equipment or device you have got at the moment? (probe: telephone/ analogue equipment such as textphone, carecall, smartphone, handheld device and computer).
   2. When did you first obtain digital devices?
   3. What made you decide which ones to buy?
   4. How useful do you find this equipment or device?
2. Could you tell me if you have internet connection at home? If not, why not? (probe: cost, no interest, don’t understand how to get connected to internet).
3. What sort of things do you usually use this equipment/device or the internet for? (probe: shopping, job search, benefits, housing, councils, health appointments, any increased use and cost and investment due to Covid19 etc?)

Understanding of the digital Training and information.

Now, we would like to find out about experience you might have had of any training to use this technology or computers:

1. Could you tell me if you have had any training to use the equipment or device you have got?
   1. How did you find out about the training?
   2. What did this training involve?
   3. Who delivered the training?
2. Where else might you go for help to use digital resources? (probe: college, courses, family and peer advice, social media, and online resources in BSL etc?)
3. Have there been any impacts on your health and wellbeing associated with quest for help? “how helpful are the support and training offered to you?” (probe: relationship with family/peer/community)

Experience with the Accessibility of equipment (including devices and tools), the systems and information.

We are interested in the benefits from using the digital technology in particular for your health and wellbeing and any challenges you have experienced. We would also want to find out what ‘good’ looks like in the technology and information accessible to D/deaf people.

1. Could you tell me about what you have used online or on your mobile phone to support you with either your physical and/or mental health? “What helps really” (Probe: text messaging or video relay services to emergency 999 or NHS111, websites, apps, social media)
2. What was good about them? (probe: what gets better. Knowing how to navigate “quickly clicking or tapping” without problems.)
3. What was not good about them? (probe: what remains stuck, hard to find what I want or “right over my head”)

Experience with the personal gain from the digital technology and information

Next, we would like to find out a bit more about what might have changed in your use of digital technology since the beginning of the pandemic including any barriers you have experienced.

We are interested in

- Results
- Impacts
- Changes
- Blocks/barriers

1. Could you tell me if your use of digital resources/equipment/internet has changed over the past two years? (probe: new phone/devices, videocall, online shopping or remote appointments)
   1. If yes, how has it changed? What has been positive/negative about the change?
   2. If not, why hasn’t it changed?
2. Do you currently experience any barriers to using digital technologies? (probe: how comfortable and safe and how confident are they using it? Or dealing with the update and changes? Any concerns about cybersecurity and how to keep the device secure?)

Digital technology is changing all the time. Now we would like to find out your views on how services could be improved in future.

1. In an ideal world, how would you want digital technology to help to improve your health and wellbeing “what is on your wish list”? (probe: think about what not good you said earlier, changes in equipment, device, connection, video consultation, online information and search etc)
2. What support would you require in order to make this better and easier for you to continue using digital technologies? (probe: training, peer support, choice between in person and online etc).
3. What can help you more? (probe: This is part of our research to actively find out new knowledge or information to create new understanding and different ways of thinking and to improve our lives – health and wellbeing of deaf people here.)
4. What have you found out more about this research or technology since we started the interview?

**Deaf people and co-production**

We are interested in understanding how and whether deaf people are involved in changes such as increased use of digital technologies in health and social care, particularly following the COVID-19 pandemic:

1. Other people might have asked you questions and completing surveys or interviews as well. Can you tell us about any experience you might have had of being part of them and about using digital technology in the delivery of health care?
   1. What was good about that experience? (probe: 1 to 1 training/tutorial, signing support, plain English questionnaire)
   2. What was not good about that experience? (probe: no communication support, lot of information in writing from the survey, choice not given).
2. Overall, how involved have you felt in the changes made to how you have accessed the health and care services since the pandemic? (probe: feeling in control and respected and listened to else at every steps or just in the beginning or at the end or not involved at all.)
3. If you were in charge, what you would do to ask for Deaf people’s view before changes are made? (probe: imagine you are one of the NHS managers and in charge.)

**Closing questions**

1. Is there anything else you would like to tell us about your experience of the topic of this interview? Or about anything else we have talked today?

**End of Interview**

- Thank participant for their time and switch off recorder.
- Complete personal demographic and other forms.
- Discuss how they feel after the interview. Ensure they have a copy of the study participant information sheet and give the debrief sheet as required.
- Check contact details for any future feedback of the results from the study.
- **OS4. Interview protocol** -
- 1) Participants approached the researcher with their expression of interest to be interviewed following their receipt of the invitation directly or via third parties (purposeful sampling).
- 2) Participants had direct contact with the researcher for their confirmation of the options regarding direct interview or with communication support, and their preferred platforms (e.g. in person at home, at a deaf centre, office base, online), plus remote platform (e.g. Teams, Zoom), and preferred language (e.g. signing, verbal, or other additional communication requirements).
- 3) Participants consent for video recording the interview sought in advance and again at the beginning of the interview.
- 4) Deaf couple consented if they would like to be interviewed separately or as a dyad.
- 5) Participants received information and instructions about the venue, online platform and the link was sent in advance.
- 6) Reasonable adjustment was made in written information from the pre-interview contact such as using bullet points, colour highlight, font size, or adding additional notes for some wording.
- 7) Interviewees supporters were involved as they requested such as their advocate, deaf centre coordinators.
- 8) Access to deaf advisors such as deaf colleagues from the DEEG, deaf colleagues within mental health services and VCSE (Voluntary, Community, and Social Enterprise) services.
- 9) Environment and equipment were rechecked on the day to optimise accessibility and that participants were prepared.
- 10) Interview structure was advised in advance and reviewed again at the beginning.
- 11) Use of both intent and prompt during the interview.
- 12) Notes were made to capture signs which were less familiar or personalised and for those vocabularies and signing agreed and used during the interview.

**OS5. Detailed Methodology**

1. Information from the bi-directional checking in field notes were referenced.

2. Video recorded data were first familiarised by the researcher as part of the reflexive thematic analysis process by watching them repeatedly and notes were made.

3. Personalised non-verbal cues (NVC) and/or non-manual features (NMF) were explored during the interview for any inconsistency, and recorded for data analysis.

4. Video recorded data were transcribed for sharing with the research team as they were not all fluent in sign language.

5. Informal notes were added alongside the transcription for any rich NMF or NCV data e.g. MOUTH, EYE, JAW, and they were in bold to signify lip patterns and facial expressions of emphasis, disagreement, affirmation etc.

6. Codes were assigned to the meaningful NMF/NCV and supported generating themes alongside the transcription.

7. Video recorded data were watched again to verify consistency in participants’ intensity of emphasis and engagement.

8. Formal notation could have been adopted with more funding for time and personnel such as a deaf co-researcher or a deaf language specialist.

| Table OS6. Themes and codes | |
| --- | --- |
| Theme and codes | Description |
| Theme 1. Lack of consideration for deaf individuals' communication preferences in digital healthcare technology | |
| *Accepting deaf identity and heritage and other challenges* | Deaf identity. Signing and experience of oralism or bilingualism. Visual learning skills. Reflecting on early childhood and upbringing and impacts from comorbidity and multiple long-term conditions on their future outlook. |
| *Social and language deprivation from experience of barriers* | Experience of and impact from being only deaf member in the family, at school and at work e.g. disconnection and isolation. Issues with language and social development. Social and language deprivation challenges. Intergenerational trauma iterates. |
| *Need for accessibility and respect* | Appropriate BSL interpreting and alternative support (face to face or remote). Accessible information. Deaf awareness delivered where deaf people are i.e. school, office, bank, GP. Dealing with threats from changes (innovation, transformation and de-investment) and fear from cybersecurity. |
| *Learning and literacy challenges* | Appropriate 1 to 1 signing/communication support e.g. interpreter, notetaker. Interaction from social, education, psychological and language deprivation. Impacts on literacy in health, English and other areas such as digital and finance. Digital literacy as part of bespoke skills training (reflection on Deaf Digital Inclusion Project and Deaf Experts by Experience group). |
| *Dilemma between deaf identity and hearing superiority* | Dependency on hearing professionals and family members. Belief that hearing people know more and know best. Questioning self-beliefs and confidence as a deaf person. |
| *Making informed choices in self-care* | Considering choices around technology - cost, data, device, reliability. Access to health and care and other supports such as employment and benefits to reinforce self-management. |
| *Personal safety associated with adverse life events* | Likelihood of interpersonal issues within deaf communities and impacts on opportunities. Exposure to bullying, abuse, trauma and exploitation. Dealing with oppression and threats. |
| Theme 2. Social connections facilitate meaningful digital health inclusion | |
| *Deaf community support and collaboration* | Valuing peer support and deaf identity, culture and heritage, ‘deafhood’ and “deaf gain” [98, 99]. Joint reflection on the impacts of covid19 pandemic and technology with deaf communities. Sharing information and updates. |
| *Reflection from the covid19 pandemic and recovery* | Deaf people’s experience with the pandemic and lockdown. Understanding of negative impacts such as face covering, changes of appointment platform. Understanding of lessons learned together with aims to promote autonomy, resilience, choice, control and to accept difficult and traumatic experience. |
| *Engagement with wider deaf communities and their supporters.* | Recognition of connection to and interface with other marginalised groups and wider communities of support (in person or online) such as advocates, carers, families and friends, and communication professionals. Issues with interpersonal safety. |
| *Tackling stigma, discrimination and injustice against deaf communities* | Identifying barriers to participation. Reflecting on experience of digital exclusion and poverty and other barriers on their adversity and trauma. Forming campaign and action groups. |
| *Promoting deaf empowerment* | Provision of deaf led training and opportunities for deaf people. Embracing peer support and celebrating deaf role models. Participating in deaf experts by experience groups. Preparation for change – visually based training and information, accessibility preference (inclusivity) and adaptability (sign language resources) and its threats. |
| *Public and patient involvement (PPI) and community engagement* | PPI with Deaf communities and their experience in wider involvement as citizens. Deaf experts by Experience Group with focus on their lived/living experience in clinical and research settings. Working with Voluntary Community and Social enterprise (VCSE) organisations with deaf communities and communication support. Feedback on experience around connectivity, reciprocity and power. |
| Theme 3. Problems with digital systems flexibility, interoperability and psychological safety | |
| *Workforce competency in working with deaf people* | The workforce is trained and prepared to work with deaf people and respond to their diverse needs even though the population is small. Workforce has access to support around technology designed and provided for deaf communities and understands their pros and cons and that they are aware of deaf people’s struggle when failing to deliver. |
| *Dilemma between deaf identity and hearing superiority* | Dependency on hearing professionals and family members. Belief that hearing people know more and know best. Questioning self-beliefs and confidence as a deaf person. |
| *Perception of psychological safety* | Deaf people's perception of their experience when they are involved and consulted. Feeling able speak up and that their contributions are equally heard. In digital transformation, deaf people are comfortable to ask, clarify, agree and disagree and suggest alternatives. |
| *Alignments between deaf people, services and systems* | NHS platforms accessible to deaf people. Shared vision between central and local technology transformation. Meeting the needs of deaf people with complex needs. Their communication, language and information preferences form part of the change. Opportunity for direct access via portal will be considered. Patients' clinical safety requirements form part of the digital health transformation. |
| Theme 4: Models of care should optimise deaf people’s independence and accessibility | |
| *Need for accessibility and respect* | Appropriate BSL interpreting and alternative support (face to face or remote). Accessible information. Deaf awareness delivered where deaf people are i.e. school, office, bank, GP. Dealing with threats from changes (innovation, transformation and de-investment) and fear from cybersecurity. |
| *Arrangement for accessible integrated care* | A co-creation approach involving all stakeholders where participants feel empowered despite their communication/language difference. Challenges and compliance with the Accessible Information Standard requirements and other reasonable adjustment requirements. Accessible data are shared and updated by end users and citizens where possible as part of their interoperability arrangement. |
| *Provision of self-directed support (personalised customised care)* | Holistic/transcultural approach in change management. Deaf people's choice and control considered to optimise independence and autonomy. Domains such as accessibility and technology are customised for round the clock care. Inputs from community, friends and family are considered. Options for additional support – flexibility and reasonable adjustments. |
| *Delivery of digitally enhanced services* | Digital and data changes are well defined and described to the deaf communities (including apps, videocall, text messaging, worn/hand-held devices, web-based, GPS/safety devices). Terms and jargon are clarified at all times. Pace of the technological initiation. Their curiosity, awareness, readiness, acceptance and compliance are evaluated. Cybersafety is monitored. Offline option is available. Understanding of non-adoption, abandonment, expansion, sustainability NASSS Framework and the implications of human factors, moral consideration and digital maturity assessment in digital health transformation [38, 39]. |
| *Learning and literacy challenges* | Appropriate 1 to 1 signing/communication support e.g. interpreter, notetaker. Interaction from social, education, psychological and language deprivation. Impacts on literacy in health, English and other areas such as digital and finance. Digital literacy as part of bespoke skills training (reflection on Deaf Digital Inclusion Project/Deaf Experts by Experience group. |
| *Impacts evaluation on health equality, equity and inclusion EDI* | Considering the diverse needs of deaf communities characterised by all protected characteristics especially, gender, age, multiple disabilities and long-term conditions, literacy and those who are unpaid carers. Implementing impact assessments and organisational culture and learning as part of the systems changes. |
| *Making informed choices in self-care* | Considering choices around technology - cost, data, device, reliability. Access to health and care and other supports such as employment and benefits to reinforce self-management. |
| *Readiness for digital and data technology* | Experience of both pros and cons. Early exposure to assistive technologies and video based social media platforms could improve their appreciation of technology and make them feel more ready to accept. |
